# Glyphosate Results Revisited: De Roos et al. Respond

**Published:** 2005-06

**Authors:** Anneclaire J. De Roos, Megan A. Svec, Aaron Blair, Jennifer A. Rusiecki, Mustafa Dosemeci, Michael C. Alavanja, Jane A. Hoppin, Dale P. Sandler

**Affiliations:** Program in Epidemiology, Fred Hutchinson Cancer Research Center and the Department of Epidemiology, University of Washington, Seattle, Washington, E-mail: deroos@u.washington.edu; Division of Cancer Epidemiology and Genetics, National Cancer Institute, National Institutes of Health, Department of Health and Human Services, Bethesda, Maryland; Epidemiology Branch, National Institute of Environmental Health Sciences, National Institutes of Health, Department of Health and Human Services, Research Triangle Park, North Carolina

The reaction of Farmer et al. regarding our article on glyphosate exposure and cancer incidence in the Agricultural Health Study (AHS) (De Roos et al. 2005) is difficult to understand given the tentative nature of our conclusions. For the most part, we found no associations with the cancers we studied, and to quote from our abstract,

Glyphosate exposure was not associated with cancer incidence overall or with most of the cancer subtypes we studied. There was a suggested association with multiple myeloma incidence that should be followed up as more cases occur in the AHS.

Despite the fact that we believe our presentation of the data was quite fair and included a lengthy discussion of possible biases affecting our results, several comments by Farmer et al. necessitate a response.

Farmer et al. had several criticisms of our review of the genotoxicity literature (De Roos et al. 2005). Although the discussion of the toxicity studies is interesting, these studies only serve as background information in our article; the epidemiologic associations between glyphosate exposure and cancer incidence we observed are the empirical result of our investigation. Criticisms of our reference to the genotoxicity literature do not, of course, alter the human data we presented. We stated in our article the conclusion of the U.S. Environmental Protection Agency (U.S. EPA 1993) and the World Health Organization (1994) that glyphosate is not mutagenic, but because that conclusion focused on the active ingredient, glyphosate, and not formulated products such as Roundup (Monsanto Company, St. Louis, MO), we also cited several studies which show potentially greater toxic effects of Roundup than glyphosate. Our article (De Roos et al. 2005) does not purport to be a comprehensive review of the toxicology literature, and because of space limitations imposed by the journal, we did not discuss several studies showing potentially toxic effects of several glyphosate-based pesticide products through disruption of cell-cycle control mechanisms, which may be relevant for cancer as well as noncancer health outcomes (Marc et al. 2002, 2004).

The fact the some of the studies we cited did not use Good Laboratory Practices is irrelevant, because this system is used primarily in analytical chemistry and contract laboratories for routine support of pesticide regulation, and is not required by any of the principal funding agencies for research studies. Studies that are submitted to the U.S. EPA to support applications for licensing pesticides are required to meet specified guidelines for record keeping, data reporting, and protocol development. These Good Laboratory Practices provide some assurance that regulators can rely on the data they review and give them the ability to perform audits as needed. Investigators who perform studies for research purposes are not required to follow these structured practices, but many may do so. Furthermore, it does not follow that work done in labs that do not strictly adhere to the U.S. EPA’s testing and reporting requirements follow “bad” laboratory practices. Quality assurance for research studies is provided by the peer-review process and by replication. This is analogous to the distinction between clinical laboratory tests performed in the context of human research and tests performed for diagnostic purposes. In order for these tests to be covered by insurers, they must be performed in laboratories approved by the Clinical Laboratory Improvement Amendments (CLIA 2005). CLIA approval assures that the test results are valid but does not address the underlying science that led to the development of the test.

In their letter, Farmer et al. used exposure information from a study by Acquavella et al. (2004) in which biomonitoring of farmers who applied glyphosate was used to determine a maximum dose calculation. The dose thresholds Farmer et al. cite as relevant for carcinogenicity are from mouse and rat models in which the active ingredient, glyphosate, was tested in feeding studies (Williams et al. 2000). Lower relevant doses may apply for Roundup and other formulated products containing glyphosate, or for glyphosate products used in combination with other active ingredients. In addition, epidemiology can provide direct information on the question of what happens in humans from more relevant routes of exposure.

Some questions were raised about the possible associations we observed between glyphosate and multiple myeloma concerning the discrepancy between the age-adjusted relative risk of 1.1 [95% confidence interval (CI), 0.5–2.4] and the relative risk adjusted for selected demographic and lifestyle variables of 2.6 (95% CI, 0.7–9.4) (De Roos et al. 2005). Farmer et al. question whether the discrepancy may be due to confounding or the selection of subjects into the more restricted analysis. This is plausible, and we discussed these issues at length in our article. The association only appeared within the subgroup with complete data on all the covariates; even without any adjustment, there was a 2-fold increased risk of multiple myeloma associated with glyphosate use among the smaller subgroup with covariate data. We acknowledged that this could be due to selection bias, effect modification, or confounding within this subgroup. We would point out, however, that confounding can be both positive and negative. The type of analysis suggested by Farmer et al., in which the data are stratified by each covariate individually in order to identify covariates for which missing data and/or adjustment made the biggest impact on the estimated relative risk, would be unreliable for such a small number of cases. Each estimate would be subject to small sample bias (Greenland 2000), which was cited by Farmer et al. as an issue with our overall estimate for myeloma. The most reliable approach will be to reanalyze the data after more cases accumulate, both to assess whether the association with myeloma persists and to further evaluate confounding and selection bias using a larger case group to support analyses. Following up initial observations with more comprehensive epidemiologic data from the AHS has been our plan since the inception of the study.
